# Serum biomarkers and changes in clinical/MRI evidence of golimumab-treated patients with ankylosing spondylitis: results of the randomized, placebo-controlled GO-RAISE study

**DOI:** 10.1186/s13075-016-1200-1

**Published:** 2016-12-28

**Authors:** Robert D. Inman, Xenofon Baraliakos, Kay-Geert A. Hermann, Jürgen Braun, Atul Deodhar, Désirée van der Heijde, Stephen Xu, Benjamin Hsu

**Affiliations:** 1 Department of Immunology, University of Toronto, Toronto, ON Canada; 2 Department of Medicine, University of Toronto, Toronto, ON Canada; 3 Department of Rheumatology, Rheumazentrum Ruhrgebiet Herne, Ruhr-University Bochum, Herne, Germany; 4 Department of Radiology, Charité Medical School, Berlin, Germany; 5 Division of Arthritis & Rheumatic Diseases, Oregon Health & Science University, Portland, OR USA; 6 Department of Rheumatology, Leiden University Medical Center, Leiden, The Netherlands; 7 Department of Biostatistics, Janssen Research & Development, LLC, Spring House, PA USA; 8 Department of Immunology, Janssen Research & Development, LLC, Spring House, PA USA

**Keywords:** ASDAS, Biologic, Monoclonal antibody, MRI, TNF inhibitor

## Abstract

**Background:**

In the present study, we evaluated relationships between serum biomarkers and clinical/magnetic resonance imaging (MRI) findings in golimumab-treated patients with ankylosing spondylitis.

**Methods:**

In the GO-RAISE study, 356 patients with ankylosing spondylitis randomly received either placebo (*n* = 78) or golimumab 50 mg or 100 mg (*n* = 278) injections every 4 weeks through week 24 (placebo-controlled); patients continuing GO-RAISE received golimumab through week 252. Up to 139/125 patients had sera collected for biomarkers/serial spine MRI scans (sagittal plane, 1.5-T scanner). Two blinded readers employed modified ankylosing spondylitis spine magnetic resonance imaging score for activity (ASspiMRI-a) and ankylosing spondylitis spine magnetic resonance imaging score for chronicity. Spearman correlations (*r*_s_) were assessed between serum biomarkers (*n* = 73) and Bath Ankylosing Spondylitis Disease Activity Index (BASDAI), C-reactive-protein (CRP)-based Ankylosing Spondylitis Disease Activity Score (ASDAS), modified Stokes Ankylosing Spondylitis Spine Score (mSASSS), and ASspiMRI scores. Serum biomarkers predicting postbaseline spinal fatty lesion development and inflammation were analyzed by logistic regression.

**Results:**

Significant, moderately strong correlations were observed between baseline inflammatory markers interleukin (IL)-6, intracellular adhesion molecule-1, complement component 3 (C3), CRP, haptoglobin, and serum amyloid-P and baseline ASDAS (*r*_s_ = 0.39–0.66, *p* ≤ 0.01). Only baseline leptin significantly correlated with ASDAS improvement at week 104 (*r*_s_ = 0.55, *p* = 0.040), and only baseline IL-6 significantly predicted mSASSS week 104 change (β = 0.236, SE = 0.073, *p* = 0.002, model *R* ^2^ = 0.093). By logistic regression, baseline leptin, C3, and tissue inhibitor of metalloproteinase (TIMP)-1 correlated with new fatty lesions per spinal MRI at week 14 and week 104 (both *p* < 0.01). Changes in serum C3 levels at week 4 (*r*_s_ = 0.55, *p* = 0.001) and week 14 (*r*_s_ = 0.49, *p* = 0.040) significantly correlated with BASDAI improvement at week 14. Baseline IL-6 and TIMP-1 (*r*_s_ = −0.63, −0.67; *p* < 0.05) and reductions at week 4 in IL-6 (*r*_s_ = 0.61, *p* < 0.05) and C3 (*r*_s_ = 0.72; *p* < 0.05) significantly correlated with week 14 ASspiMRI-a improvement.

**Conclusions:**

Extensive serum biomarker multiparametric analyses in golimumab-treated patients with ankylosing spondylitis demonstrated few correlations with disease activity or MRI changes; IL-6 weakly correlated with radiographic progression.

**Trial registration:**

ClinicalTrials.gov identifier: NCT00265083. Registered on 12 December 2005.

**Electronic supplementary material:**

The online version of this article (doi:10.1186/s13075-016-1200-1) contains supplementary material, which is available to authorized users.

## Background

Magnetic resonance imaging (MRI) is now an established outcome instrument in the assessment of treatment for ankylosing spondylitis (AS), based on results of randomized, placebo-controlled studies. Such studies have shown that MRI can provide an objective assessment of response to tumor necrosis factor (TNF) inhibition in patients with AS [1, 2].

In the GO-RAISE trial of golimumab in AS (ClinicalTrials.gov identifier: NCT00265083), patients with active AS showed significant improvement in signs and symptoms after treatment with golimumab [3] that were maintained through 2 years [4]. In the MRI substudy of GO-RAISE, golimumab significantly reduced MRI-detected spinal inflammation, and these observed improvements correlated with improvement in signs and symptoms of disease [5]. Data from observational studies suggest that long-term treatment with TNF antagonists, especially if started early, may inhibit radiographic progression [6]. In another study, a clear causal relationship between the level of inflammation as assessed by the Ankylosing Spondylitis Disease Activity Score (ASDAS) and radiographic progression has been established [7]. Moreover, a moderate relationship between spinal inflammation detected by MRI and syndesmophyte formation has been proven [8–11]. Therefore, we conducted additional analyses of data from the GO-RAISE trial to further assess potential relationships between serum biomarkers of inflammation and clinical measures of disease activity, as well as with spinal inflammation and fatty lesions detected by MRI.

## Methods

### Patients and study design

The details of the GO-RAISE study have been described previously [3, 4]. The protocol was reviewed and approved by the institutional review board or independent ethics committee at each site of this multicenter trial. (See Acknowledgements section below for further details.) All patients provided written informed consent. The study was conducted at 57 sites in the United States, Canada, Europe, and Asia. The MRI substudy was conducted at ten sites with the capability and willingness to participate. All patients enrolled at participating substudy sites were included in the MRI substudy.

GO-RAISE patients were adults with definite AS (for ≥3 months) according to modified New York criteria, a Bath Ankylosing Spondylitis Disease Activity Index (BASDAI) score ≥4 (0- to 10-point scale), a total back pain score ≥4 on a visual analogue scale (0- to 10-cm scale), and an inadequate response to current or previous nonsteroidal anti-inflammatory drugs (NSAIDs). Patients were randomly assigned in a 1:1.8:1.8 ratio to receive subcutaneous injections every 4 weeks of placebo, golimumab 50 mg, or golimumab 100 mg. Randomization was stratified by investigational site and screening C-reactive protein (CRP) concentration. Patients were allowed to continue stable doses of methotrexate, sulfasalazine, hydroxychloroquine, corticosteroids, and NSAIDs throughout study participation.

At week 16, patients who achieved <20% improvement from baseline in both the total back pain and morning stiffness scores entered into double-blinded early escape, such that patients in the placebo group received golimumab 50 mg, patients in the golimumab 50 mg group received 100 mg, and patients in the golimumab 100-mg group had no change to their study therapy. The placebo-controlled portion of the study ended at week 24, following which all patients who had been receiving placebo injections crossed over to receive golimumab 50 mg. Injections continued to be administered subcutaneously every 4 weeks through week 252, and the last study assessments for the period covered by the current analyses were at week 208.

### Biomarker evaluations

Serum samples collected at weeks 0, 4, and 14 of the GO-RAISE trial were tested by Rules Based Medicine (Austin, TX, USA) and Pacific Biomarkers (Seattle, WA, USA) for selected markers using Luminex (Luminex, Austin, TX, USA) and enzyme-linked immunoassay platforms, respectively. The extensive list of candidate biomarkers implicated from previous studies (Additional file 1: Table S1) [12] was selected for correlation analyses with imaging and clinical endpoints, including leptin, interleukin (IL)-6, tissue inhibitor of metalloproteinase (TIMP)-1, complement component 3 (C3), intracellular adhesion molecule (ICAM)-1, CRP, haptoglobin, and serum amyloid-P.

### Disease activity and progression evaluations

Clinical assessments of disease activity included the BASDAI [13] and the ASDAS employing CRP [14] determined at weeks 0, 14, and 104. MRI was employed to assess inflammation and structural changes in the spine. Serial spine 1.5-T MRI scans of the cervical, thoracic, and lumbar spine regions in the sagittal plane were acquired at baseline, week 14, and week 104 [5]. Two qualified readers who were blinded to treatment information, patient identity, and chronology of the images independently scored each sequence using modified versions of the ankylosing spondylitis spine magnetic resonance imaging score for activity (ASspiMRI-a) and the Ankylosing spondylitis spine magnetic resonance imaging score for chronicity (ASspiMRI-c) [15, 16]. ASspiMRI-a scoring of each vertebral unit (VU) was previously described [5]. For the modified ASspiMRI-c score, each VU was scored as follows: 0 (normal, no lesions), 1 (minor fatty degeneration), 2 (much fatty degeneration), 3 (one or two syndesmophytes), 4 (more than two syndesmophytes), 5 (vertebral bridging with <25% of disc length and/or bridging of one side), or 6 (vertebral fusion with >25% of disc length and/or bridging of both sides). If a fatty lesion was present in a VU scored 3–6, it was noted. Lateral view radiographs of the cervical and lumbar spine regions were obtained at weeks 0, 104, and 208 and scored by two trained readers blinded to treatment group and chronology using the modified Stokes Ankylosing Spondylitis Spine Score (mSASSS) method [17], as described previously [18].

### Data analysis

All analyses of MRI data collected through week 104 employed observed data; missing data were not imputed. Of the unique serum biomarkers tested in this trial, 73 had <80% of values below the lower limit of quantitation at baseline and are included in the analyses described herein (Additional file 1: Table S1).

A total of 4940 analyses were performed to determine Spearman correlation coefficients describing relationships between serum biomarkers at weeks 0, 4, and 14 and subsequent efficacy endpoints, including ASDAS, BASDAI, ASspiMRI-a, ASspiMRI-c (all determined at weeks 0, 14, and 104), and mSASSS (determined at weeks 0, 104, and 208). Correlations at baseline were assessed on the basis of all patients, whereas those at follow-up time points were assessed according to randomized treatment groups (placebo versus golimumab). For all postbaseline assessments, changes from baseline in biomarkers were used. Only correlations found to be statistically significant after Bonferroni multiplicity-of-testing adjustment are reported herein.

Relationships between four biomarker levels (IL-6, leptin, TIMP-1, and C3) and changes from baseline to week 104 in mSASSS were also assessed using a generalized linear model. In this model, mSASSS scores were adjusted for VUs with fat indicated via ASspiMRI-c score at baseline, because the presence of fatty lesions on MRI images is a known contributor to radiographic (mSASSS) progression [19, 20] and because these markers affect adipocytes.

Logistic regression analyses were also conducted to assess the influence of baseline and earlier (week 14) changes in biomarker levels on the development of new fatty lesions on a patient level (yes or no, i.e., not detected by MRI at baseline but detected at week 14 or week 104). In such analyses, all patients were combined, with no adjustment for treatment group or multiplicity of testing. The resulting odds ratios and 95% confidence intervals were determined.

## Results

### Patient analysis

One hundred thirty-nine patients had serum biomarker data, and 98 of these patients at 10 sites with MRI capability participated in the GO-RAISE MRI substudy. The vast majority of randomized patients (299 [84.0%] of 356) had pre- and posttreatment data obtained for the GO-RAISE radiographic substudy. Of these, up to 139 patients had sera collected for biomarker evaluations, and up to 125 underwent spine MRI scans. As reported previously, the demographic and baseline characteristics for the MRI substudy patients were generally consistent with those of the overall GO-RAISE patient population [5].

### Correlations between baseline biomarker levels and baseline disease activity

At baseline among all patients with serum biomarker assessments (*n* = 98–139), significant and moderately strong correlations were observed between ASDAS and levels of the following serum biomarkers of inflammation: IL-6, ICAM-1, haptoglobin, serum amyloid-P, and C3 (*r*_s_ = 0.39–0.59, all *p* ≤ 0.01) (Table 1). As would be expected, ASDAS correlated with CRP (*r*_s_ = 0.66, *p* = 0.00) (Table 1). With respect to baseline serum markers and baseline spinal inflammation (ASspiMRI-a score) or structural lesions (ASspiMRI-c score), no significant correlations were observed.

**Table 1:** Correlations between baseline serum biomarker levels and baseline ASDAS

	Baseline ASDAS Spearman correlation coefficient (*r*_s_)		
Baseline serum biomarker	Patients (*n*)	*r*_s_	Bonferroni-adjusted *p* value
IL-6	132	0.59	0.000
ICAM-1	139	0.39	0.011
C3	98	0.53	0.000
CRP	98	0.66	0.000
Haptoglobin	98	0.46	0.010
Serum amyloid-P	98	0.50	0.001

*Abbreviations: ASDAS* Ankylosing Spondylitis Disease Activity Score employing C-reactive protein, *CRP* C-reactive protein, *C3* Complement component 3, *ICAM-1* Intracellular adhesion molecule-1, *IL* Interleukin

### Correlations between baseline biomarker levels and subsequent changes in measures of disease activity, radiographic progression, and spinal inflammation

Baseline leptin level was the only significant biomarker correlated with ASDAS clinical improvement at week 104 of golimumab treatment (*r*_s_ = 0.55, *p* = 0.040). Higher baseline levels of IL-6 (*r*_s_ = −0.63, *p* = 0.009) and TIMP-1 (*r*_s_ = −0.67, *p* = 0.044), but not baseline CRP, significantly correlated with subsequent improvement in ASspiMRI-a score (i.e., greater reduction) from baseline to week 14 of golimumab treatment (Table 2). All other correlations tested between baseline biomarkers and subsequent changes in measures of disease activity or spinal inflammation yielded insignificant relationships.

**Table 2:** Significant correlations between serum biomarkers and Ankylosing Spondylitis Disease Activity Score, Bath Ankylosing Spondylitis Disease Activity Index, and ankylosing spondylitis spine magnetic resonance imaging score for activity changes among golimumab-treated patients

Serum biomarker, time, number of golimumab-treated patients	Spearman correlation coefficient (*r*_s_); Bonferroni-adjusted *p*-value with respective outcome
	Change in ASDAS from baseline to week 104
Leptin	
Baseline, *n* = 58	0.55; 0.040
	Change in BASDAI from baseline to week 14
C3	
Change from baseline to week 4, *n* = 76	0.55; 0.001
Change from baseline to week 14, *n* = 76	0.49; 0.040
	Change in ASspiMRI-a score from baseline to week 14
IL-6	
Baseline, *n* = 48	−0.63; 0.009
Change from baseline to week 4, *n* = 48	0.61; 0.022
Change from baseline to week 14, *n* = 48	0.59; 0.043
TIMP-1	
Baseline, *n* = 35	−0.67; 0.044
C3	
Change from baseline to week 4, *n* = 35	0.72; 0.005

*Abbreviations: ASDAS* Ankylosing Spondylitis Disease Activity Score employing C-reactive protein, *ASspiMRI-a* Ankylosing spondylitis spine magnetic resonance imaging score for activity, *BASDAI* Bath Ankylosing Spondylitis Disease Activity Index, *C3* Complement component 3, *IL* Interleukin, *TIMP-1* Tissue inhibitor of metalloproteinase-1

Using a generalized linear model to assess the ability of baseline serum leptin, C3, TIMP-1, and IL-6 levels to predict subsequent change in mSASSS, only baseline serum IL-6 levels were found to significantly predict change in mSASSS at week 104 (β = 0.236, SE = 0.073, *p* = 0.002, model *R* ^2^ = 0.093; data not shown). Results of logistic regression analyses indicated that baseline levels of inflammatory and metabolic markers leptin, C3, and TIMP-1 were statistically significant factors in the development of new fatty lesions in the spine at both week 14 and week 104 of golimumab treatment. Change from baseline to week 14 in TIMP-1 also significantly factored into the development of fatty lesions at week 104 (Table 3). Whereas most of these predictive relationships were very weak, those between higher baseline C3 levels and the seven- to eightfold lower risk of subsequent fatty lesion development were of note.

**Table 3:** Logistic regression analysis of serum biomarkers and fatty lesion development visualized by magnetic resonance imaging

	Fatty lesion development, odds ratio (95% confidence interval); *p* value	
Serum biomarker, time	Week 14 (*n* = 125)	Week 104 (*n* = 117)
Leptin		
Baseline	0.92 (0.87–0.98); *p* = 0.005	0.86 (0.78–0.95); *p* = 0.004
C3		
Baseline	0.13 (0.04–0.44); *p* = 0.001	0.15 (0.04–0.61); *p* = 0.008
TIMP-1		
Baseline	1.02 (1.01–1.03); *p* = 0.005	1.02 (1.01–1.04); *p* = 0.001
Change from baseline to week 14	0.98 (0.97–1.00); *p* = 0.030	0.96 (0.94–0.98); *p* = 0.000

*Abbreviations: C3* Complement component 3, *TIMP-1* Tissue inhibitor of metalloproteinase-1

Results of a separate generalized linear model used to evaluate potential interactions between biomarkers and baseline patient characteristics show that IL-6, leptin, TIMP-1, and C3 influenced mSASSS progression independently of demographic factors of age, sex, and human leukocyte antigen B27 positivity (data not shown).

### Correlations between posttreatment biomarker levels and posttreatment measures of disease activity and spine inflammation

Reductions in C3 observed from baseline to week 4 (*r*_s_ = 0.55, *p* = 0.001) and week 14 (*r*_s_ = 0.49, *p* = 0.040) significantly correlated with improvement from baseline to week 14 in the BASDAI score. Similarly, improvement from baseline to week 4 in serum IL-6 (*r*_s_ = 0.61, *p* = 0.022) and C3 (*r*_s_ = 0.72, *p* = 0.005) levels, as well as improvement from baseline to week 14 in serum IL-6 levels (*r*_s_ = 0.59, *p* = 0.043), correlated significantly with improvement in ASspiMRI-a scores from baseline to week 14 of golimumab treatment (Table 2). All other correlations tested between posttreatment biomarker levels and posttreatment measures of disease activity or spinal inflammation yielded insignificant relationships.

## Discussion

The search for serum biomarkers of prognostic utility in AS reflects, in part, the need for objective measures of disease activity and response to treatment, as well as the unresolved issues of pathogenesis in the disease. The structured nature of a randomized controlled trial provides more clinical uniformity in treatment regimen and outcome measures than is possible in real-world observational studies. In the present study, significant and moderately strong correlations were observed between ASDAS and levels of IL-6, ICAM-1, haptoglobin, serum amyloid-P, and C3. As expected, similar results were observed for CRP. These correlations suggest possible roles for these biomarkers in AS-related inflammation. Baseline serum IL-6 and TIMP-1, as well as reductions in IL-6 and C3, correlated with reduction in ASspiMRI-a scores. Of note, ASDAS has been shown to correlate well with ASspiMRI-a scores, and weakly with BASDAI change, among golimumab-treated patients with AS [5].

Decreases in TIMP-1 at week 14 correlated with fatty lesion development at weeks 14 and 104. The biological basis of local inflammation being associated with reparative processes in spine and local inflammation being reflected as new fatty lesions have yet to be defined, and TIMP-1 would be a candidate factor for future studies. Previously described predictors, such as insulin, matrix metalloproteinase-3, vascular endothelial growth factor, or bone resorption markers, did not significantly correlate with clinical or imaging outcomes.

Fatty lesion development (fat metaplasia) is thought to reflect postinflammation “repair” and may be a precursor of new bone formation. When assessed by logistic regression, baseline levels of inflammatory and metabolic markers leptin, C3, and TIMP-1 were statistically significant factors in the risk of development of new fatty lesions in the spine at both week 14 and week 104 of golimumab treatment, but only the relationship between higher baseline C3 levels and a seven- to eightfold decreased risk of subsequent fatty lesion development offers potential clinical utility. Both leptin and C3, the precursor of C3adesArg acylation-stimulating protein, are involved in triglyceride uptake into adipocytes and homeostasis of adipose tissue. Deficiency of leptin is associated with lipodystrophy and resistance to obesity, whereas increased C3 is linked to increased central obesity and insulin resistance [21]. Furthermore, inflammatory cytokines such as TNF-α and IL-6 stimulate production of leptin and C3, and decreased levels of the receptor for C3adesArg, C5L2 [21, 22]. Together with these properties of leptin and C3, our finding that increased baseline levels of leptin and C3 were correlated with decreased risk of vertebral fatty lesion development suggests that these are key regulators of local adipogenesis within bone tissue in AS. Although Li and colleagues also demonstrated underexpression of C3 in patients with AS (*n* = 6) relative to healthy volunteers (*n* = 6) in a preliminary search for disease-associated proteins in the sera of patients with AS [23], additional studies are needed to confirm these findings.

### Limitations

A limitation of the present study is the large number of analytes, which precludes analyses such as principal component analysis or supervised cluster analysis. To guard against identifying a false-positive relationship between the large number of analytes assessed and disease activity outcomes, we employed the Bonferroni correction in the statistical modeling to account for multiplicity of testing. The crossover to active anti-TNF agent after a relatively short placebo period complicates interpretation of “placebo group” imaging data at weeks 104 and 208. These analyses are also limited by the fact that golimumab doses were combined and not assessed individually; however, given that no dose-response relationship was observed between golimumab 50 mg and 100 mg when analyzed by randomized groups at week 104 [4], the doses were combined to maximize the patient numbers in the golimumab arm. Despite this approach, there remained few patients on whom to base correlation analyses for radiographic progression and development of fatty lesions. This is an unresolved issue and should be the focus of future research in this field. Additionally, IL-6 levels can be influenced by therapeutic corticosteroid use via suppression of endogenous cortisol levels [24, 25] and also by visceral fat secretion [26]; our analyses did not adjust for these potential confounders in assessing relationships between IL-6 and MRI-detected spinal inflammation and clinical measures of disease activity.

## Conclusions

An extensive serum biomarker analysis in golimumab-treated patients with AS demonstrated that few biomarkers showed correlation with disease activity or MRI changes in multiparametric analysis. Although IL-6 weakly correlated with radiographic progression, other biomarkers found in prior studies to be significantly associated with AS imaging changes were not confirmed.

## Additional file

Additional file 1: Table S1. Serum biomarker panel. Table provides a detailed list of the serum biomarkers evaluated. (DOCX 24 kb)

## Abbreviations

AS: Ankylosing spondylitis
ASDAS: Ankylosing Spondylitis Disease Activity Score
ASspiMRI-a: Ankylosing spondylitis spine magnetic resonance imaging score for activity
ASspiMRI-c: Ankylosing spondylitis spine magnetic resonance imaging score for chronicity
BASDAI: Bath Ankylosing Spondylitis Disease Activity Index
C3: Complement component 3
CRP: C-reactive protein
ICAM-1: Intracellular adhesion molecule-1
IL: Interleukin
MRI: Magnetic resonance imaging
mSASSS: Modified Stokes Ankylosing Spondylitis Spine Score
NSAID: Nonsteroidal anti-inflammatory drug
*r*_s_: Spearman correlation coefficient
TIMP-1: Tissue inhibitor of metalloproteinase-1
TNF: Tumor necrosis factor
VU: Vertebral unit
