# Supplementary material for: Serum biomarkers and changes in clinical/MRI evidence of golimumab-treated patients with ankylosing spondylitis: results of the randomized, placebo-controlled GO-RAISE study
Source: Arthritis Res Ther. 2016 Dec 28;18:304. doi: 10.1186/s13075-016-1200-1 (PMC5192572; doi:10.1186/s13075-016-1200-1)
Supplement: Additional file 1: Table S1. — Serum biomarker panel. Table provides a detailed list of the serum biomarkers evaluated. (DOCX 24 kb) [file 13075_2016_1200_MOESM1_ESM.docx]

**ADDITIONAL FILE 1.**

| **Table S1. Serum biomarker panel** |
| --- |
| 1. Adiponectin |
| 1. Alpha1_Antitrypsin |
| 1. Alpha2_Macroglobulin |
| 1. AlphaFetoprotein |
| 1. Apolipoprotein_A1 |
| 1. Apolipoprotein_CIII |
| 1. Apolipoprotein_H |
| 1. Beta2_Microglobulin |
| 1. Bone Alkaline Phosphatase |
| 1. BrainDerived_Neurotrophic_Factor |
| 1. CD40 |
| 1. CD40_Ligand |
| 1. COL 2-3/4C Long Neoepitope |
| 1. C_Reactive_Protein |
| 1. Cancer_Antigen_125 |
| 1. Cancer_Antigen_199 |
| 1. Carcinoembryonic_Antigen |
| 1. Complement_3 |
| 1. Deoxypyridinoline |
| 1. EGF |
| 1. ENA78 |
| 1. ENRAGE |
| 1. Eotaxin |
| 1. Factor_VII |
| 1. Ferritin |
| 1. Fibrinogen |
| 1. GCSF |
| 1. Glutathione_STransferase |
| 1. Growth_Hormone |
| 1. Haptoglobin |
| 1. Hyaluronic Acid |
| 1. ICAM-1/ICAM1 |
| 1. IGF1 |
| 1. IL-6 Serum (ELISA) |
| 1. IL-8 |
| 1. IL-16 |
| 1. IL-18 |
| 1. IL-1ra |
| 1. IL-7 |
| 1. IL-8 |
| 1. IgA |
| 1. IgE |
| 1. IgM |
| 1. Insulin |
| 1. Leptin |
| 1. Lipoprotein_a |
| 1. MCP1 |
| 1. MDC |
| 1. MIP1alpha |
| 1. MIP1beta |
| 1. MMP-3 (Serum) |
| 1. Myeloperoxidase |
| 1. Myoglobin |
| 1. N Terminal Propeptide of Type 1 Procollagen (P1NP) |
| 1. Osteocalcin |
| 1. PAI1 |
| 1. Prostate_Specific_Antigen_Free |
| 1. Prostatic_Acid_Phosphatase |
| 1. Pyridinoline |
| 1. RANTES |
| 1. SGOT |
| 1. SHBG |
| 1. Serum_Amyloid_P |
| 1. Stem_Cell_Factor |
| 1. TIMP1 |
| 1. TNF-Alpha Serum (ELISA) |
| 1. TNF_RII |
| 1. TNFalpha |
| 1. Thyroid_Stimulating_Hormone |
| 1. Thyroxine_Binding_Globulin |
| 1. VCAM1 |
| 1. VEGF/VEGF (serum) |
| 1. von_Willebrand_Factor |
